# Correspondence

**Published:** 1878-05

**Authors:** 


### Miscellaneous.

### CORRESPONDENCE.

PROF. J. TAFT, D. D. S.

EDITOR DENTAL REGISTER:—I sent to your address, a copy of Zahnærztlicher (Dental) Almanach for 1878, by Adolf Petermann, D. D. S., a graduate of Philadelphia Dental College, and now practicing dentistry in Frankfort a. M. Germany.

The Almanach of this year, includes the names and post-office addresses of dental practitioners in Austria and Hungary. The list of dentists in the German Empire has been carefully revised and completed, and is presented in the same elegant style as heretofore.

The arrangement of names, etc., is so much better than anything attempted in this country, that it deserves special mention.

First. The names of dental practitioners, alphabetically arranged, together with the professional status of the individual. If a graduate, the college which conferred the degree—whether of medicine, surgery, dental surgery or dental medicine and the year of graduation; the city or town, street and number of office or residence.

Second. An alphabetically arranged register of cities and towns, the number of inhabitants and the surnames of dentists practicing therein.

Next, a statistical review, according to which there are four hundred and fifty-nine practicing dentists in the German Empire, six of which are females—among the latter, Maria Goubert, D. D. S., (Ohio College of Dental Surgery, 1872); Elise Von Heyden, D. D. S., (Baltimore College of Dental Surgery, 1876); Henriette Hirschfeld, D. D. S., (Pennsylvania College of Dental Surgery, 1869).

These four hundred and fifty-nine dentists have a working-field of 504,628.5 square Kilometers, and among a population of 42,757,812 inhabitants. In Austria and Hungary, a territory of 623,966.6 square Kilometers, and a population of 35,904,435. We find recorded one hundred and twenty-two dentists. In both Empires, with a population of 77,662,247, the almanach records five hundred and eighty-one dentists.

Among these, there are fifty-two D. D. S., graduates of American Dental Colleges, two of which, (Dr. Joseph Linderer, Baltimore College of Dental Surgery, 1851, and Dr. Wm. L. Eastlake, Ohio College of Dental Surgery, 1873), received the Degree *Honoris Causa*, twenty-seven Doctors *Med.*, (two of which *Honoris Causa*), twenty-four Doctors *Med.* and *Chir.*, thirteen Doctors *Philos.*, four Doctors *Chir.*, one M. D. M., (Harvard University Dental Department), and one Doctor *Chem.*

In addition to this, a list of dental periodicals, published in Germany, France, Italy, England and America with their respective ages. The Dental Register in its thirty-second year, heads the list; L'Art Dentaire, twenty-two years old, comes next, to be followed by the Journal of Dental Science in its twenty-first year, and the Dental Cosmos in its twentieth year, and so on all through. Likewise a list of national, state and local associations and societies of Europe and of many of this country; together with the time and place of meeting, and the names of presiding officers.

Last, but not least, a correspondence between the author and several gentlemen (twelve in number) who sported the title of "Doctor of Dentistry," "Doctor of Dental Surgery," etc., claiming that they had obtained their diplomas,—some from the "Philadelphia of Medicine and Surgery" and others from the "Livingston University of America."

This correspondence is spicy and of weight, and has probably been the means of stopping, or at least, very much diminished the traffic in bogus diplomas.

For this bold and fearless exposure of a shameless swindle, the author deserves the thanks and acknowledgments of all right minded practitioners of America, as it relieves our colleges of any imputation of dishonorable transactions in granting diplomas to those who had never been students within their walls. It does more perhaps in establishing and upholding a proper professional status than could be obtained by legislative enactment.

Permit me to remark here, that if the several state societies would inaugurate a similar measure, publish a yearly dental calendar with the proceedings of their respective societies, (as Ohio has done and does now) I think much good would result therefrom; provided however, it were done in the same thorough and impartial manner as it has been done by Dr. Petermann.

The little book needs no praise, it is simply elegant, and speaks for itself. Lithographs, print, paper and binding, all first class.

I understand it is for sale at the dental depots. All those who have an idea of going to Europe with the intention of practicing dentistry, will find information contained therein, scarcely to be obtained elsewhere.

Every dentist should procure a copy of this little work, if for no better reason than to show their appreciation of the vast amount of labor, pluck and perseverance of the author.

Members of our state society will recollect what it cost the society to procure the simply approximately correct list of dentists of our one state, which was done some three years since. You who have been revising this list every year, know how much labor and vexation it entails upon those who undertake such enterprises. You also know how much good has resulted therefrom.

Ohio may therefore claim the honor of having been the pioneer of inaugurating a similar undertaking to that of Dr. Petermann. F. H. R.

